# Prevalence and Clinical Correlates of Suicide Attempts Among Young Patients With Major Depressive Disorder With Subclinical Hypothyroidism: A Cross‐Sectional Study

**DOI:** 10.1002/brb3.71765

**Published:** 2026-09-23

**Authors:** Na Wang, Guoshuai Luo, Qianqian Wei, Jing Long, Xiangyang Zhang

**Affiliations:** ^1^ Psychological Outpatient Department Tianjin Anding Hospital Mental Health Center of Tianjin Medical University Tianjin China; ^2^ Laboratory of Biological Psychiatry Institute of Mental Health Tianjin Anding Hospital Mental Health Center of Tianjin Medical University Tianjin China; ^3^ Hefei Fourth People's Hospital Anhui Mental Health Center Affiliated Psychological Hospital of Anhui Medical University Hefei China

**Keywords:** first episode, major depressive disorder, subclinical hypothyroidism, suicide attempts, young adults

## Abstract

**Objective:**

Depression frequently co‐occurs with subclinical hypothyroidism (SCH), but its role in suicide risk among young patients with major depressive disorder (MDD) remains unclear. We investigated suicide attempts (SAs) among a large cohort of young, first‐episode drug‐naïve (FEDN) patients with MDD and SCH.

**Method:**

A total of 917 FEDN patients with MDD (351 males and 566 females; mean age = 24.7 ± 5.4 years, range = 18–35 years) were recruited. Sociodemographic, clinical data, and thyroid function parameters were collected from all patients. Anxiety, depression, psychotic symptoms, and overall clinical severity were assessed using the Hamilton Anxiety Rating Scale (HAMA), Hamilton Depression Rating Scale (HAMD), Positive and Negative Syndrome Scale Positive Subscale, and Clinical Global Impression (CGI) scale, respectively.

**Results:**

Young MDD patients had an increased prevalence of SCH (56.8%). Among young MDD cases with SCH, SAs were associated with age, marital status, illness duration, and HAMA score; for HAMA score, B = 0.167, OR = 1.182, 95% CI: 1.062–1.315, and *p = *0.002. SAs were also associated with thyroid stimulating hormone (TSH) (B = 0.315, OR = 1.371, 95% CI: 1.180–1.592, *p < *0.001), thyroid peroxidase antibody (TPOAb) (B = 0.004, OR = 1.004, 95% CI: 1.002–1.006, *p < *0.001) and CGI score. Combining HAMA, TSH, and CGI scores differentiated those who attempted suicide from those who did not (area under the curve = 0.792). HAMD, HAMA, TPOAb, TSH, illness duration, age, and marital status were independently related to the number of SAs (*p* < 0.001).

**Conclusions:**

Young FEDN patients with MDD showed a high prevalence of SCH, and SAs were common among those with comorbid SCH. Clinical characteristics and thyroid function indicators were independently associated with SAs, highlighting the importance of thyroid screening and suicide‐risk assessment in young patients with MDD.

## 1 Introduction

Suicide refers to a self‐inflicted death with evidence of intent to die, whereas attempted suicide (SA) refers to a nonfatal act with intent to end one's life (Klonsky et al. 2016). Suicide is often associated with major depressive disorder (MDD) and is a major cause of death among young adults (Ong et al. 2021). A survey in the United States showed that young adults (18–29 years) exhibited an obvious increased rate of SAs compared with adults over 30 years of age (Crosby et al. 2011). In China, the prevalence of SAs among patients with MDD has been reported to range from 7.3% to 48.4% (Dong et al. 2018). However, data regarding suicide and SAs among young individuals with depression remain limited.

MDD usually develops in early adulthood, and its high recurrence and persistence can lead to disability throughout the lifespan (Davey and McGorry 2019; Gathright et al. 2017). MDD is also one of the most important psychiatric disorders associated with suicide and SAs among young adults (Melhem et al. 2019; Pitman et al. 2012). Compared with older adults, young people exhibit different risk factors for SAs and are more likely to engage in impulsive and violent self‐destructive behaviors (Lesage et al. 1994; Page et al. 2014). In addition, SAs have broader health implications; for example, Shanahan et al. (2016) reported that SAs in early life were associated with an increased risk of cardiovascular health problems in the late twenties. Consequently, considerable research has focused on identifying factors associated with suicide risk in young patients with MDD, including abnormalities of the hypothalamic–pituitary–adrenal stress‐response system (Orsolini et al. 2020; H. Li et al. 2020).

Thyroid hormones exert various effects on the central nervous system, and subclinical hypothyroidism (SCH) remains one of the common concomitant symptoms of MDD (Charnsil and Pilakanta 2017). SCH is biochemically defined as elevated thyroid stimulating hormone (TSH) concentrations in the presence of normal free thyroxine (FT4) levels (Cooper 2001; Peeters 2017). Although the mechanisms linking MDD and thyroid dysfunction remain unclear, previous studies have consistently demonstrated a higher prevalence of SCH among patients with MDD than among individuals without depression (Karakatsoulis et al. 2021; Tang et al. 2019; Zhao et al. 2021). Furthermore, anxiety and depressive symptoms appear to be more severe in MDD patients with thyroid dysfunction (Gulseren et al. 2006; Siegmann et al. 2018), and MDD patients with comorbid SCH have been reported to exhibit a longer illness duration and higher positive symptom scores (Kang et al. 2023).

Several studies have also suggested a potential relationship between thyroid dysfunction and suicidal behavior. Shen et al. (2019) reported elevated serum levels of thyroid peroxidase antibodies (TPOAb), thyroglobulin antibodies (TgAb), and TSH in MDD patients with SAs compared with those without SAs. In addition, previous studies have reported associations between abnormalities of the hypothalamic–pituitary–thyroid (HPT) axis, including altered thyrotropin‐releasing hormone (TRH) activity, and suicidal behavior (Mann and Currier 2007; Duval et al. 2017). However, findings remain inconsistent, as some studies have reported no significant association between SCH and subsequent suicide risk (Kørner et al. 1987; Toloza et al. 2021). Therefore, whether SCH contributes to the risk of SAs in patients with MDD remains unclear.

Age may be an important factor in this relationship because both thyroid function and suicide‐related behaviors have been shown to vary across the lifespan (Zhang et al. 2023). Younger patients with MDD often exhibit prominent vegetative and physiological symptoms, including changes in energy, weight, appetite, and sleep (Rice et al. 2019), suggesting that endocrine dysfunction may play a particularly important role in this population. Supporting this possibility, Hirtz et al. (2022) reported that HPT axis function in adolescents with MDD differed from that observed in adults. Nevertheless, evidence regarding the association between SCH and depression in younger populations remains inconclusive. Kim et al. (2018) found no significant relationship between SCH and depression in a large prospective cohort of young and middle‐aged adults, while other studies involving adolescents and young adults also failed to identify a significant association between SCH and depressive symptoms (Hirtz et al. 2022). Dorn et al. (1996) reported an association between abnormal thyroid function and negative behaviors in adolescents with depression but did not specifically examine SAs.

Taken together, existing evidence suggests that MDD, SCH, age, and suicidal behavior may be interrelated. However, the prevalence of SAs among young MDD patients with comorbid SCH and the clinical factors associated with SAs in this population remain insufficiently investigated. Since thyroid function may be influenced by antidepressant treatment and illness chronicity (Sauvage et al. 1998), we recruited a large cohort of first‐episode, drug‐naïve MDD patients aged 18–35 years. The aims of the current study were to investigate the prevalence of SAs in young MDD patients with SCH and to identify the clinical and biochemical factors associated with SAs in this population.

## 2 Materials and Methods

### 2.1 Subjects

We performed a cross‐sectional study in which young outpatients were recruited consecutively from the psychiatric outpatient department of the First Hospital of Shanxi Medical University. During the course of our research, we followed the Declaration of Helsinki and received approval from the Institutional Review Board (IRB) of the First Hospital of Shanxi Medical University (Reference: 2016‐Y27; approved on September 7, 2016). Each enrolled patient received comprehensive information regarding the research, was provided with information sheets, and signed informed consent forms.

Patients were included if they met the following criteria: (1) were aged 18–35 years and were of Han Chinese; (2) met the MDD criteria of the Structured Clinical Interview for DSM‐IV (SCID) as assessed by two experienced psychiatrists; (3) had a 17‐item Hamilton Rating Scale for Depression (HAMD) score ≥ 24; (4) had not previously used antidepressants or antipsychotic treatment; and (5) had not received thyroxine therapy or other targeted medications.

Participants were excluded if they had (1) any Axis I psychiatric disorder besides MDD; (2) physical comorbidities that may affect thyroid function; (3) history of substance abuse; (4) pregnancy or breastfeeding; or (5) inability to understand, refusal to accept, or difficulty in completing research procedures.

### 2.2 Clinical Measures

Sociodemographic data, including gender, age, marital status, and education, were acquired from all participants (C. H. Lin et al. 2014). In addition, we collated the time of disease onset, duration of disease, and body mass index (BMI).

The assessment of depression was based on the 17‐item HAMD. Representative items include “depressed mood,” “feelings of guilt,” and “work and activities.” This scale contains eight items rated on a 5‐point scale (0: absent, 4: severe), with nine items ranging from 0 (absent) to 2 (symptom‐specific severity descriptor). Depression severity was evaluated by the HAMD score: ≤ 7 = not depressed (NDP), ≥ 8 = depressed (DP), ≤ 17 = mild‐to‐moderate depression, and ≥ 24 = severe depression (Luo et al. 2023). The Chinese‐language version of the HAMD scale was verified in 1985 (Zhu 1985).

The assessment of anxiety symptoms was based on the Hamilton Anxiety Rating Scale (HAMA), which includes 14 items rated on a 5‐point Likert scale (0: not present; 4: severe). Representative items include “anxious mood,” “tension,” and “insomnia.” The Chinese version of the HAMA scale was verified in 1986 (G. X. Lin 1986). Patients with an overall score of > 29 are classified as having severe anxiety (Konstantakopoulos et al. 2013).

The Positive Symptoms Scale, one of the subscales of the Positive and Negative Syndrome Scale (PANSS), was utilized to assess and quantify positive psychotic symptoms in the study population. Representative items include “delusions,” “hallucinatory behavior,” and “conceptual disorganization.” Responses were measured using a 7‐point Likert scale, where 1 indicated “none” and 7 indicated “extremely severe.” Thus, the total PANSS positive subscale score ranged from 7 to 49. Kay et al. (1987) reported that psychosis was defined as an overall positive subscale score ≥ 15.

The Clinical Global Impression (CGI) scale was initially designed as an assessment tool for psychopharmacology studies (Guy 1976) and has been established as a valid tool for assessing mood, anxiety, and psychiatric disorders (Montoya et al. 2011). Unlike symptom‐specific rating scales, the CGI‐S provides an overall clinician‐rated assessment of illness severity. The CGI‐S employs a score of 1 (normal) to 7 (most severe) to assess the clinician's impression of the current severity of illness. The Chinese adaptation of the CGI‐S was established as valid in 1984 (Wu et al. 2012).

Two qualified psychiatrists underwent training in the administration of these rating scales before the study commenced. After repeated assessments, their inter‐observer correlation coefficients for the HAMA, HAMD, and PANSS scores were all > 0.8. These psychiatrists were blinded to the clinical profile of each patient.

In this study, we followed the view of Klonsky et al. (2016) in that SAs are a self‐imposed act or behavioral tendency with the intention of killing oneself, whether or not the tendency or behavior causes actual harm. Typically, SAs were measured by interviewing the patients (Joiner et al. 2003; Posner et al. 2007). Patients were questioned about previous SAs over the course of their lifetime, and those who answered “yes” were deemed suicide attempters. In this study, only actual SAs were recorded; suicidal ideation, suicidal plans, and behavioral tendencies without an overt suicidal act were not considered SAs. The researcher subsequently asked further questions about the frequency of attempts, the mode of attempted suicide, and the exact date. When patient responses were ambiguous, additional information was collected through interviews with family members, relatives, or close friends.

### 2.3 SCH Measurement

Participants were asked to fast overnight, and had their blood samples taken from 6 to 8 a.m. Serum levels of free triiodothyronine (FT3), FT4, TPOAb, TgAb, and TSH were assessed using a Roche C6000 electro‐chemiluminescent immunoassay analyzer (Roche Diagnostics, USA). In this study, we defined TSH between 0.27 and 4.20 mIU/L as the normal range. The normal ranges for FT3, FT4, TPOAb, and TgAb were 3.10–6.8 pmol/L, 10–23 pmol/L, 0–34 IU/L, and 0–115 IU/L, respectively.

SCH was defined as TSH > 4.2 mIU/L with FT4 levels within the reference range (X. Li et al. 2017). SCH was further classified as mild (TSH < 10 mIU/L) and severe SCH (TSH ≥ 10 mIU/L) according to serum TSH levels (Lang et al. 2020).

### 2.4 Statistical Analysis

All statistical tests were conducted with SPSS 26.0. (International Business Machines Corp). Categorical variables were presented as percentages, and differences were analyzed using cross‐tabulation tables or chi‐squared analyses. For continuous variables, the Kolmogorov–Smirnov (K–S) test was first employed to determine whether they were normally distributed. In this study, none of the continuous variables were normally distributed (all *p* < 0.01) and they did not conform to the normal distribution despite log or square root transformations being performed. In this case, we utilized the Mann–Whitney *U* test to analyze differences between different groups. We then selected variables that differed significantly in binary comparisons as independent variables to be included in a logistic regression model. Differences at the *p* < 0.05 level were deemed significant.

To deeply explore the strength of the correlation among the relevant variables, this study constructed a binary logistic regression model. In the regression analyses, we assessed the odds ratio (OR) and its 95% confidence interval (CI). The Wald test was utilized to evaluate the statistical significance of each variable in the model. Given that this study involves the simultaneous analysis of multiple clinical indicators, there is a problem of multiple comparisons, which may raise the probability of Type I error (false positive). To address this, we applied Bonferroni correction to the significance level.

The area under the receiver operating characteristic curve (AUC) was employed to assess different the discriminatory power of each classification model. In this study, we calculated the AUC values for all parameters that were significant for suicide in the logistic regression model. AUC values > 0.7 were accepted (Hosmer et al. 2013). Microsoft Excel 2019 (Microsoft, Redmond, WA, USA) was used to prepare figures.

## 3 Results

In total, 917 first‐episode and medication‐naïve patients were enrolled in this study, of whom 351 were males and 566 were females (Table 1). The average age of patients was 24.7 ± 5.43 years (range: 18–35 years). A total of 481 patients (52.5%) were unmarried. The average duration of education was 13.9 ± 2.65 years (range: 9–19 years). Overall, 93.8% of the patients had attained senior high school education or higher. The age of onset was 24.6 ± 5.39 years. The average duration of illness was 5.0 ± 3.34 months (range: 0.5–21 months). Of the participants, 10.8% (*n* = 99) had severe anxiety symptoms, and 83 (9.1%) had psychotic symptoms. The prevalence of SCH (mild and severe SCH combined) was 57.0% (523/917) in the total sample, and 179 (19.5%) patients had attempted suicide.

**TABLE 1:** Demographics and clinical characteristics of patients with and without SCH.

	Young MDD without SCH (*n* = 394)	Young MDD with SCH (*n* = 523)	*χ* ^2^ or Δd ± SD	*p*
Gender (M/F)	141/253	210/313	1.813	0.178
Married (yes) (%)	48.2%	47.0%	0.127	0.722
Education (years)	13.71	14.04	−0.33 ± 0.176	0.068
Age	24.57	24.77	−0.19 ± 0.374	0.590
Duration of MDD (months)	4.47	5.35	−0.88 ± 0.221	0.001
Age of onset	24.48	24.66	−0.18 ± 0.371	0.630
Severe anxiety (HAMA > 29) (%)	5.3%	14.9%	21.433	< 0.001
Psychotic symptoms (yes), (%)	4.1%	12.8%	20.899	< 0.001
Suicide attempts (yes), (%)	11.7%	25.4%	27.064	< 0.001
Repeated suicide (yes), (%)	2.5%	4.2%	1.857	0.173
Suicide by violence (yes), (%)	6.6%	15.5%	17.225	< 0.001
BMI (kg/m^2^)	23.90	24.61	−0.71 ± 0.130	0.001
CGI	5.72	6.07	−0.35 ± 0.047	0.001
HAMD	28.71	31.25	−2.54 ± 0.173	0.001
HAMA	20.07	21.13	−1.06 ± 0.213	0.001
PANSS (positive subscore)	7.74	9.46	−1.72 ± 0.250	0.001
FT3 (pmol/L)	4.89	4.98	−0.09 ± 0.047	0.053
FT4 (pmol/L)	16.81	16.75	−0.06 ± 0.197	0.756
TGAb (abnormal) (%)	8.6%	22.9%	32.954	< 0.001
TPOAb (abnormal) (%)	11.9%	32.1%	51.052	< 0.001

Abbreviation: BMI, body mass index; CGI, Clinical Global Impression of Severity Scale; FT3, free triiodothyronine; FT4, free thyroxine; HAMA, Hamilton Anxiety Rating Scale; HAMD, Hamilton Rating Scale for Depression; MDD, major depressive disorder; PANSS, Positive and Negative Syndrome Scale; SCH, subclinical hypothyroidism.

### 3.1 Prevalence of SAs in Young MDD Patients With Comorbid SCH in Comparison With Those With No SCH

Compared to young MDD patients with no SCH, young MDD patients with SCH had a prolonged course of illness, greater scores on HAMD, HAMA, and PANSS positive subscale, greater levels of CGI and BMI, and a higher probability of TgAb and TPOAb abnormalities (all *p* ≤ 0.001) (Table 1). The OR between SCH and non‐SCH patients was 3.11 (95% CI: 1.886–5.139; *χ* ^2^ = 21.433; *p* < 0.000) for severe anxiety symptoms, and 3.471 (95% CI: 1.979–6.090; *χ* ^2^ = 20.899; *p* < 0.000) for psychotic symptoms.

The proportion of patients who had attempted suicide was 11.7% (*n* = 45) in patients without SCH and 25.4% (*n* = 133) in SCH patients (*χ* ^2 = ^27.064, *p* < 0.001, OR = 2.580, 95% CI: 1.791–3.717). Binary logistic regression analysis showed that, after adjusting for marital status, age at onset, MDD duration, BMI, HAMD, HAMA, and psychosis, SCH patients were 1.684 times more likely to attempt suicide than those without SCH (OR: 1.684, 95%CI: 1.086–2.610, Wald *χ* ^2^ = 5.420, *p* < 0.05).

Moreover, MDD cases with SCH used more violent SA methods (non‐SCH: 6.6%; SCH: 15.5%, *χ* ^2^ = 17.225, *p *< 0.001, OR = 2.594, 95% CI: 1.633–4.121) than those without SCH. Violent SA methods included self‐destruction by explosion, crashing a car, jumping off a building, shooting themselves, self‐immolation, suicide by cutting the neck, suicide with sharp or blunt instruments, and hara‐kiri. Non‐violent SA methods predominantly involved drug ingestion, poisoning, drowning, and hanging.

### 3.2 Clinical Features and Biochemical Markers of Suicide Attempters Versus Non‐Attempters Among Young MDD Cases With SCH

Of the total sample of 917 young MDD patients, 523 had comorbid SCH. Table 2 shows the details of the personal characteristics and clinical variables compared between suicide attempters and non‐attempters. The two groups differed significantly on the HAMD, HAMA, PANSS positive subscale, and CGI scores (all *p* < 0.001) Compared with non‐attempters, suicide attempters exhibited higher serum TSH levels and were more likely to have severe SCH, and abnormal serum TPOAb and TgAb levels (all *p* < 0.001). The OR between suicide attempters and non‐attempters was 3.50 (95%CI: 2.126–5.762; *χ* ^2 = ^26.217; df = 1; *p* < 0.000) for severe anxiety, 3.996 (95%CI: 2.354–6.781; *χ* ^2 = ^29.124; df = 1; *p* < 0.000) for psychosis, 3.040 (95%CI: 1.967–4.699; *χ* ^2 = ^26.322; df = 1; *p* < 0.000) for TgAb abnormality, and 2.905 (95%CI: 1.931–4.370; *χ* ^2 = ^27.255; df = 1; *p* < 0.000) for TPOAb abnormality. All differences remained significant after Bonferroni correction.

**TABLE 2:** Demographics and clinical characteristics between young MDD with comorbid SCH with and without SA.

	Young MDD comorbid SCH		F/ *χ* ^2^ / *z*	*p*
	Without SA (*n* = 390)	With SA (*n* = 133)		
Age	24.53	25.46	−1.689	0.091
Gender (M/F)	160/230	50/83	0.486	0.486
Married (yes) (%)	47.9%	44.4%	0.512	0.474
Education (years)	14.10	13.86	−0.838	0.402
Age of onset	24.43	25.35	−1.692	0.091
Illness duration (months)	5.21	5.77	−0.753	0.452
HAMD	30.73	32.77	−7.318	< 0.001
HAMA	20.33	23.48	−8.487	< 0.001
Psychotic positive score	8.67	11.80	−6.173	< 0.001
CGI	5.91	6.56	8.230	< 0.001
BMI (kg/m^2^)	24.67	24.44	−1.616	0.106
TSH (mIU/mL)	6.18	7.82	−8.426	< 0.001
Severe SCH (yes) (%)	2.3%	12.8%	23.032	< 0.001
FT3 (pmol/L)	5.00	4.89	−1.480	0.139
FT4 (pmol/L)	16.71	16.87	−0.372	0.701
TgAb (abnormal) (%)	17.4%	39.1%	26.322	< 0.001
TPOAb (abnormal) (%)	25.9%	50.4%	27.255	< 0.001

Abbreviations: BMI, body mass index; CGI, Clinical Global Impression of Severity Scale; FT3, free triiodothyronine; FT4, free thyroxine; HAMA, Hamilton Anxiety Rating Scale; HAMD, Hamilton Rating Scale for Depression; MDD, major depressive disorder; SCH, subclinical hypothyroidism; TgAb, thyroglobulin antibody; TPOAb, thyroid peroxidase antibody; TSH, thyroid stimulating hormone.

### 3.3 Risk Factors for SAs in Young MDD Cases With SCH

Next, we performed logistic regression analyses with SAs as the outcome variable and gender, age, marital status, disease duration, depression severity, anxiety severity, psychotic symptoms, BMI, CGI, TSH levels, FT3 levels, FT4 levels, TgAb levels, and TPOAb levels as independent variables. These analyses showed that age (*B *= 0.121, OR = 1.129, CI: 1.046–1.217, *p = *0.003), marital status (*B* = 1.239, OR = 3.453, CI: 1.530–7.791, *p = *0.003), illness duration (*B* = 0.072, OR = 1.075, CI: 1.002–1.153, *p = *0.044), HAMA score (*B* = 0.167, OR = 1.182, 95% CI: 1.062–1.315, *p = *0.002), TSH levels (*B* = 0.315, OR = 1.371, 95% CI: 1.180–1.592, *p* < 0.001), TPOAb levels (*B* = 0.004, OR = 1.004, 95% CI: 1.002–1.006, *p* < 0.001), and CGI (*B* = 0.674, OR = 1.962, 95% CI: 1.276–3.017, *p = *0.002,) were specific risk factors for SAs in young MDD cases with SCH (Table 3).

**TABLE 3:** Factors associated with suicide attempts in young MDD patients with SCH.

	*B*	Wald	*p*	OR	95% CI
Age	0.121	9.809	0.002	1.129	1.046–1.217
Marital status	1.239	8.908	0.003	3.453	1.530–7.791
Disease duration	0.072	4.058	0.044	1.075	1.002–1.153
HAMA	0.167	9.389	0.002	1.182	1.062–1.315
TSH	0.315	17.081	0.000	1.371	1.180–1.592
CGI	0.674	9.423	0.002	1.962	1.276–3.017
TPOAb level	0.004	20.261	0.000	1.004	1.002–1.006

Abbreviations: CGI, Clinical Global Impression of Severity Scale; HAMA, Hamilton Anxiety Rating Scale; MDD, major depressive disorder; SCH, subclinical hypothyroidism; TPOAb, thyroid peroxidase antibody; TSH, thyroid stimulating hormone.

**FIGURE 1:**
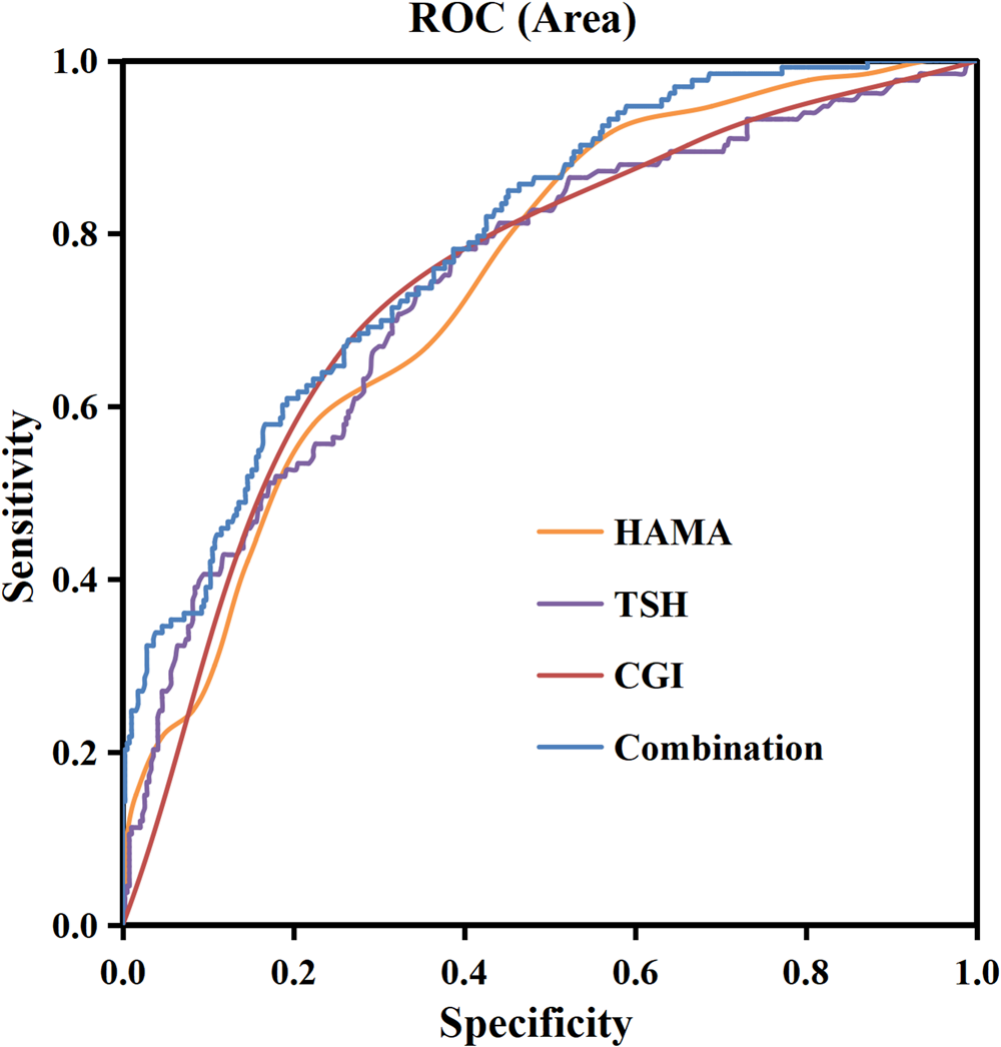
The discriminatory capacity of related factors for distinguishing between patients with and without suicide attempts in young MDD patients comorbid with SCH. The area under the curve for HAMA score, TSH, CGI score, and the combination of these three factors were 0.745, 0.744, 0.725, and 0.792, respectively. CGI, Clinical Global Impression of Severity Scale; HAMA, Hamilton Anxiety Rating Scale; MDD, major depressive disorder; SCH, subclinical hypothyroidism; TSH, thyroid stimulating hormone.

In addition, AUC values were calculated for significant variables identified by the binary logistic regression analyses. The discriminatory power of the HAMA alone as a dependent variable model was 0.745, represented by AUC values of 0.744 (TSH), 0.725 (CGI), 0.653 (TPOAb), 0.549 (age), 0.522 (disease duration), and 0.482 (marital status). The combined model consisted of variables with AUC values > 0.7. The AUC value for the combined model was 0.792 and was more significant than the individual models. These findings imply that combining HAMA score, TSH level, and CGI score helped to differentiate suicide attempters from non‐attempters (*p* < 0.0001, 95%CI: 0.749–0.834) (Figure 1). The optimal cut‐off values for the four models were determined using the Youden index (see Table 4). Among them, the TSH‐only model showed the highest sensitivity (73.7%), while the combined model demonstrated the highest specificity (80.8%). These findings suggest that the TSH‐only model has greater sensitivity for screening SAs among young patients with MDD comorbid with SCH, making it more suitable for identifying individuals at potential risk. In contrast, the combined model, with higher specificity, is more appropriate for confirming the presence of SAs and identifying high‐risk patients.

**TABLE 4:** Comparison of predictive performance of different models for suicide risk in first‐episode drug‐naïve MDD patients with comorbid SCH.

Model	AUC (95% CI)	Optimal cut‐off	Sensitivity (%)	Specificity (%)	Youden Index
Combined model	0.792 (0.749–0.834)	0.346	60.9	80.8	0.417
HAMA‐only model	0.745 (0.699–0.791)	22.5	57.9	77.7	0.356
TSH‐only model	0.744 (0.695–0.794)	6.47	73.7	65.6	0.393
CGI‐only model	0.725 (0.749–0.834)	6.5	65.4	75.1	0.405

Abbreviations: CGI, Clinical Global Impression of Severity Scale; HAMA, Hamilton Anxiety Rating Scale; MDD, major depressive disorder; SCH, subclinical hypothyroidism; TSH, thyroid stimulating hormone.

Risk factors for violent SA methods were also investigated in young MDD cases with SCH. Gender, age, marital status, disease duration, depression severity, anxiety severity, psychotic symptoms, BMI, CGI, TSH levels, FT3 levels, FT4 levels, TgAb levels, and TPOAb levels was included independent variables. The analysis results show that illness duration (*B* = 0.120, OR = 1.128, CI: 1.041–1.221, *p = *0.003), HAMA score (*B* = 0.156, OR = 1.169, CI: 1.034–1.322, *p = *0.013), psychotic positive score (*B* = −0.083, OR = 0.920, CI: 0.856–0.989, *p = *0.025), TSH (*B* = 0.247, OR = 1.280, CI: 1.082–1.514, *p = *0.004), TgAb (*B* = −0.002, OR = 0.998, CI: 0.997–1.000, *p = *0.018), TPOAb (*B* = 0.004, OR = 1.004, CI: 1.003–1.006, *p = *0.000) and CGI (*B* = 0.550, OR = 1.734, CI: 1.057–2.845, *p = *0.029) were all identified as significant factors in the regression model (Table 5).

**TABLE 5:** Factors associated with violent suicide attempt methods in young MDD patients with SCH.

	*B*	Wald	*p*	OR	95% CI
Disease duration	0.120	8.727	0.003	1.128	1.041–1.221
HAMA	0.156	6.206	0.013	1.169	1.034–1.322
Psychotic symptoms	−0.083	5.038	0.025	0.920	0.856–0.989
TSH	0.247	8.254	0.004	1.280	1.082–1.514
TgAb	−0.002	5.552	0.018	0.998	0.997–1.000
TPOAb	0.004	21.732	0.000	1.004	1.003–1.006
CGI	0.550	4.747	0.029	1.734	1.057–2.845

Abbreviations: CGI, Clinical Global Impression of Severity Scale; HAMA, Hamilton Anxiety Rating Scale; MDD, major depressive disorder; SCH, subclinical hypothyroidism; TgAb, thyroglobulin antibody; TPOAb, thyroid peroxidase antibody; TSH, thyroid stimulating hormone.

### 3.4 Correlation of the Number of SAs With Clinical, Metabolic, and TSH Markers in Young MDD Cases With SCH

Finally, multivariate regression analyses were employed to analyze the factors influencing the number of SAs. Analyses showed that marital status (Beta = −0.144, *t* = −2.331, *p = *0.020), age (Beta = 0.195, *t* = 3.078, *p = *0.002), disease duration (Beta = 0.124, *t* = 3.034, *p = *0.003), HAMD (Beta = 0.173, *t* = 2.779, *p = *0.006), HAMA (Beta = 0.159, *t* = 2.588, *p = *0.010), TSH (Beta = −0.143, *t* = 2.994, *p = *0.003), and TPOAb (Beta = 0.223, *t* = 5.008, *p* < 0.000) were all independently related to the number of SAs in young MDD cases with SCH.

## 4 Discussion

This research focused on young FEDN MDD cases. This research represents, to our knowledge, the first observational investigation with a large sample to assess the prevalence and clinical correlates of SAs in young MDD cases with SCH. We found that the rate of comorbid SCH in young MDD patients was as high as 56.8%. In young MDD cases with SCH, the prevalence of SAs (25.4%) was 1.684‐fold higher compared to young MDD patients with no SCH (11.7%). Among young MDD cases with SCH, SAs were associated with HAMA scores, CGI scores, as well as the levels of TPOAb and TSH. Combining HAMA, TSH, and CGI scores was able to differentiate between suicide attempters and non‐attempters with an AUC of 0.792. In addition, both TPOAb and TSH were related to violent SA methods and the number of SAs in young MDD patients with SCH.

The relationships between abnormal thyroid function and depressive symptoms have been explored for over 60 years, and it has been suggested that thyroid hormones are potential biomarkers for depression (Young et al. 2016). The prevalence of SCH is known to be higher in depressed patients than within the overall population (Peeters 2017; Haggerty et al. 1993). Only a few studies have focused on young patients with depression and SCH. Hirtz et al. (2021) suggested that the prevalence of SCH and of thyroid autoimmunity in young cases with depression is increased, although there was no specific concomitant rate was provided. Herein, we identified a high prevalence (56.8%) of SCH among young MDD cases.

The comorbid rates of MDD and SCH derived from different studies vary widely. A multicenter study by the European Group for the Study of Resistant Depression (GSRD) determined that the point prevalence of comorbid hypothyroidism in MD patients was 13.2% (Fugger et al. 2018). In another study, Ojha et al. (2013) found that thyroid dysfunction was common (21.4%) in depressed patients, and that 11.4% of patients with depression met the criteria for SCH. A more recent meta‐analysis that included 12,315 MDD patients demonstrated that the prevalence of SCH was 13.8% (Loh et al. 2019). Zhou, Ma, et al. (2021) noted that 24.62% of patients with MDD in China had abnormal levels of thyroid hormones. Saxena et al. (2000) reported TSH abnormalities in 54.5% of patients with treatment‐naïve unipolar depression.

The prevalence of SCH is known to be influenced by diverse factors, including age, sex, BMI, race, dietary iodine intake, and the cutoff serum TSH concentration employed to define the disorder (Cooper and Biondi 2012). Different geographic regions, economic factors, lifestyles, and dietary habits may also influence the comorbid rates of SCH and depression (Teng et al. 2006). In addition, patients with first‐onset untreated depression may have more severe abnormal physiological markers (Arabska et al. 2018). For example, Jia et al. (2005) recruited 26 patients with first‐onset and untreated MDD and found that biochemical and thyroid function abnormalities may be present early in the course of MDD. These findings suggest that abnormal thyroid function, or SCH, is more likely in patients with first‐episode and unmedicated depression.

The higher rate of comorbid SCH in young patients is a cause for concern. A missed diagnosis of SCH may not only lead to mood cycling or depression but may also result in a delayed response to treatment (Karakatsoulis et al. 2021). Furthermore, our analyses found that young MDD cases with SCH attempted suicide more often than those with no SCH. Previous studies have demonstrated similar findings. For instance, Sanna et al. (2014) found that depressed patients with thyroid disease were 3.85‐fold more likely to develop suicidal ideation than those without thyroid disease. In a registry‐based Danish study, Heiberg‐Brix et al. (2019) found that patients with Hashimoto's thyroiditis, with a significant increase in TPOAb and TgAb as the most meaningful diagnostic indicators, had a higher frequency of suicidal deaths than controls with normal thyroid function.

It is known that multiple factors influence SAs in MDD cases. Our research provides enhanced evidence that various clinical features and biochemical indicators, including HAMA score, TSH, TgAb, and CGI score, were related to SAs in young MDD cases with SCH. Gili et al. (2019) identified affective disorders as a risk factor for SAs in people aged 12–26 years in a broader systematic review. However, these authors did not report the severity of symptoms. In studies that did not target young MDD patients with SCH, results consistently indicated that anxiety severity elevates the number of SAs in MDD patients. Sareen et al. (2005) identified a 4.15‐fold higher risk of SAs in MDD cases with concomitant anxiety disorders when compared to those with no anxiety disorders. A further meta‐analysis of 28 articles by Hawton et al. (2013) showed that severe anxiety was related to a higher risk of SAs in patients with MDD by 1.59‐fold. It is worth noting that several studies have reported that depression severity can also predict SAs in MDD cases (Hoertel et al. 2017), although this association was not observed in young FEDN MDD cases with SCH. One possible explanation is that anxiety symptoms may reflect heightened psychological distress, agitation, and arousal that are more closely related to suicidal behavior than overall depressive symptom severity. In addition, because all participants had severe MDD (HAMD score ≥ 24), the restricted range of depressive symptom severity may have reduced its ability to distinguish between patients with and without SAs. However, this interpretation remains speculative and warrants further investigation. Furthermore, analyses of violent SA methods and the number of SAs did not exert a marked effect on the severity of depression.

Our findings clearly showed that SAs were related to the serum levels of TPOAb and TSH in young MDD cases with SCH. Moreover, both TSH and TPOAb were related to violent SA methods and the number of SAs. Cross‐sectional research has shown that patients with higher serum levels of TSH tended to attempt suicide (Berlin et al. 1999). Zhang et al. (2023) found that TSH was remarkably higher in adolescent MDD cases with SAs than those without. A Chinese study on MDD patients by Zhou, Ren, et al. (2021) also reported that serum TPOAb and TSH levels were related to SAs. Importantly, serum TgAb and TPOAb levels are known biomarkers of autoimmune thyroiditis (Karakatsoulis et al. 2021). We found that SAs correlated with abnormal serum concentrations of TgAb and TPOAb, implying that the immune system may be closely related to SAs in young MDD cases with SCH. However, the findings regarding violent SA methods and the number of violent SA methods should be interpreted with caution, as these subgroup analyses were based on relatively small sample sizes and may therefore be less stable than the analyses of overall SAs.

Indeed, neurobiological studies partially explain the roles of thyroid function in SAs. It has long been reported that thyrotropin hyporesponsiveness to TRH stimulation is common in patients with MDD (Karakatsoulis et al. 2021); this mechanism affects the serum levels of thyrotropin and is strongly related to SAs. The relationships between suicidal behavior and the HPT system have been brought to the attention of researchers, and relevant studies have provided evidence supporting the involvement of thyroid hormones in the regulation of key neurotransmitters (5‐hydroxytryptamine and norepinephrine) associated with suicidal behavior (Toloza et al. 2021). Jokinen et al. (2008) argued that in depressed patients, a lower TSH response to TRH was related to violent SAs and suicidal intent. Banki et al. (1984) identified a significant difference in the TSH response to TRH between violent and non‐violent suicide attempters. Duval et al. (2017) also found that non‐violent suicide attempters demonstrated a higher TSH‐TRH response than in non‐violent suicide attempters than violent suicide attempters. FT3 and FT4 may also be associated with SAs in depressed patients (Toloza et al. 2021; Peng et al. 2018). However, we did not find an obvious effect of FT3 and FT4 on suicidal ideation. Further studies should be conducted to explore the specificity of young MDD cases with SCH.

The present study has certain limitations that warrant consideration. First, we conducted a cross‐sectional study design; thus, our analyses can only show that relevant factors were associated with SAs but cannot confirm a causal relationship between them. Second, given that the sample comprised young FEDN patients with MDD, further studies are needed to confirm these findings across different age ranges and clinical settings. Third, certain confounding factors that play a key role in SAs, such as smoking, alcohol and drug abuse, personality defects, and family history of psychiatric disorders; these factors were not considered in the present study. Fourth, there are many risk factors for suicide among young MDD patients with SCH; however, this study considered only a limited number of relevant variables, thus having limitations in variable selection strategy (Chowdhury and Turin 2020) and lacking exploration of interactions among these variables. Fifth, although blood sampling was standardized to the morning period to reduce the influence of circadian variation and fasting status on thyroid‐related measurements, potential selection bias related to recruitment from a clinical population of young individuals with MDD should be considered. However, participation was not restricted by the early‐morning sampling schedule, as eligible participants who were unable to complete blood collection on the day of assessment were scheduled for sampling within 1 week. Finally, as no healthy control group was included, the results must be interpreted with caution and considered preliminary. Considering these limitations as well as the limitations from previous studies, longitudinal research is needed to clarify the impact and progression of thyroid dysfunction and autoimmunity in early‐onset depression.

## 5 Conclusion

Our findings demonstrated an extremely high rate of comorbid SCH in young FEDN MDD patients, amounting to 56.8%, thus suggesting the necessity of routine thyroid function monitoring in young MDD patients. Anxiety severity, CGI, the serum levels of TPOAb and TSH, age, marital status, and illness duration were identified as risk factors for SAs in young MDD cases with SCH. Furthermore, illness duration, the HAMA score, positive subscale score, TSH levels, TgAb levels, TPOAb levels, and CGI emerged as risk factors for violent SA methods. In addition, marital status, disease duration, the severity of depression, anxiety severity, TPOAb level, and TSH level were independently related to the number of SAs. Therefore, it is recommended that young MDD patients undergo routine thyroid function monitoring and that their suicide risk be taken seriously. Future studies should investigate the prevalence of antibodies directed against the central nervous system and examine the impact of immunosuppressive therapy on treatment‐resistant depression in young adults. Such research may contribute to more effective treatment strategies and improved quality of life for young patients with depression.

## Author Contributions

**Na Wang**: conceptualization, methodology, formal analysis, Writing – original draft, visualization. **Guoshuai Luo**: writing – original draft, conceptualization, methodology, writing – review and editing. **Qianqian Wei**: data curation, writing – review and editing. **Jing Long**: writing – review and editing, validation. **Xiangyang Zhang**: investigation, resources, project administration, writing – review and editing, funding acquisition, supervision.

## Funding

This study was supported by the National Natural Science Foundation of China (Reference: 81973759), the National Key R&D Program of China (Reference: 2020YFC2003100), and the Hospital‐level Project by Tianjin Anding Hospital (Reference: ADYKHT2021012). These sources of funding had no further role in this study design, in the data collection and analysis, in the writing of the report, or in the decision to submit this paper for publication.

## Ethics Statement

The study was approved by the Institutional Review Board (IRB) of the First Hospital of Shanxi Medical University (2016‐Y27) and conducted in accordance with the Declaration of Helsinki.

## Consent

Informed consent was obtained from all individuals included in this study.

## Conflicts of Interest

The authors declare no conflicts of interest.

## Data Availability

The data that support the findings of this study are available from the corresponding author upon reasonable request.
